# Induction of ferroptosis by natural products in non-small cell lung cancer: a comprehensive systematic review

**DOI:** 10.3389/fphar.2024.1385565

**Published:** 2024-05-01

**Authors:** Qiang Zhang, Yuting Xia, Feiyan Wang, Dongfeng Yang, Zongsuo Liang

**Affiliations:** Key Laboratory of Plant Secondary Metabolism and Regulation of Zhejiang Province, College of Life Sciences, Zhejiang Sci-Tech University, Hangzhou, China

**Keywords:** non-small cell lung cancer, ferroptosis, natural products, cancer treatment, drug development

## Abstract

Lung cancer is one of the leading causes of cancer-related deaths worldwide that presents a substantial peril to human health. Non-Small Cell Lung Cancer (NSCLC) is a main subtype of lung cancer with heightened metastasis and invasion ability. The predominant treatment approaches currently comprise surgical interventions, chemotherapy regimens, and radiotherapeutic procedures. However, it poses significant clinical challenges due to its tumor heterogeneity and drug resistance, resulting in diminished patient survival rates. Therefore, the development of novel treatment strategies for NSCLC is necessary. Ferroptosis was characterized by iron-dependent lipid peroxidation and the accumulation of lipid reactive oxygen species (ROS), leading to oxidative damage of cells and eventually cell death. An increasing number of studies have found that exploiting the induction of ferroptosis may be a potential therapeutic approach in NSCLC. Recent investigations have underscored the remarkable potential of natural products in the cancer treatment, owing to their potent activity and high safety profiles. Notably, accumulating evidences have shown that targeting ferroptosis through natural compounds as a novel strategy for combating NSCLC holds considerable promise. Nevertheless, the existing literature on comprehensive reviews elucidating the role of natural products inducing the ferroptosis for NSCLC therapy remains relatively sparse. In order to furnish a valuable reference and support for the identification of natural products inducing ferroptosis in anti-NSCLC therapeutics, this article provided a comprehensive review explaining the mechanisms by which natural products selectively target ferroptosis and modulate the pathogenesis of NSCLC.

## 1 Introduction

**FIGURE 1:**
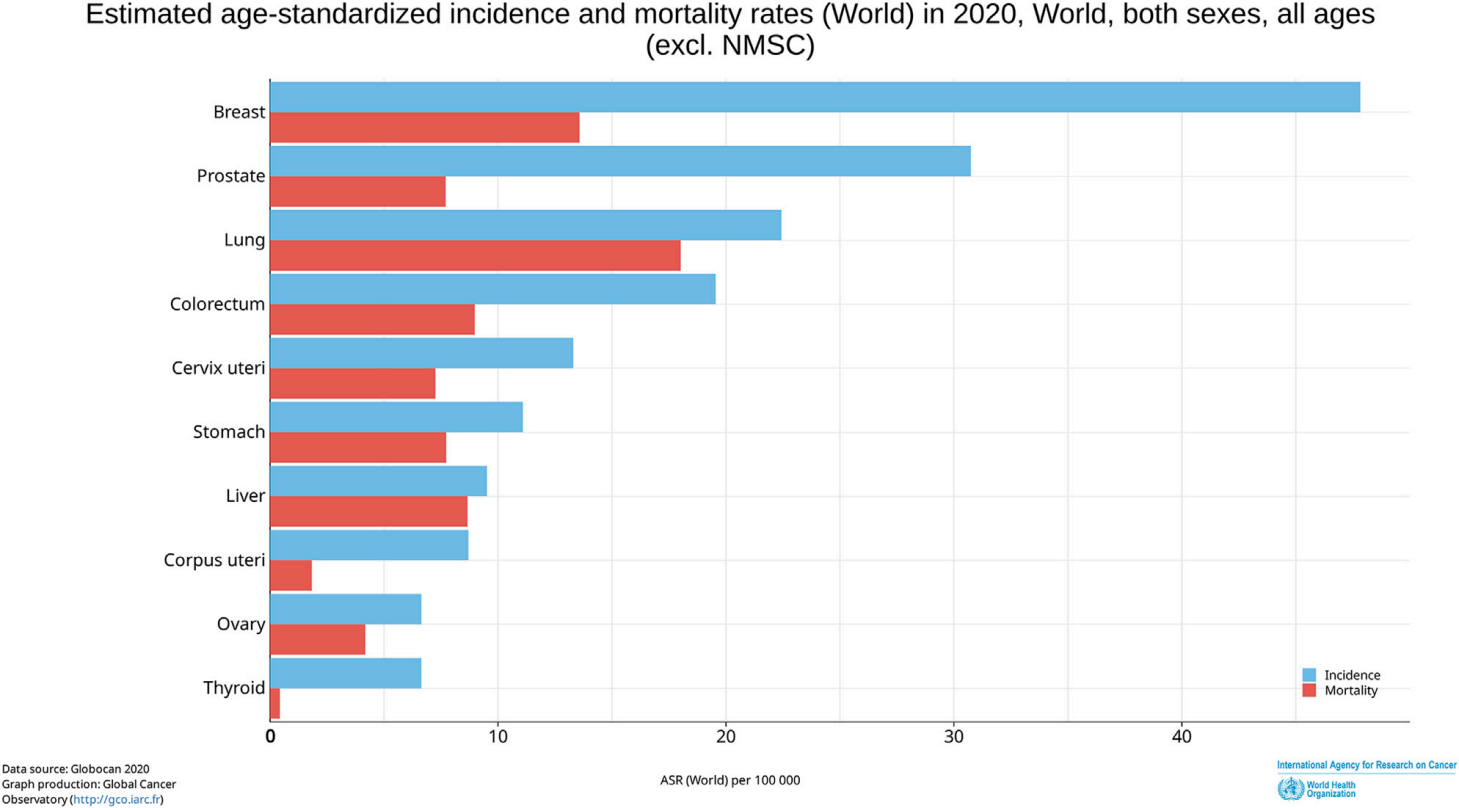
Estimated age-standardized incidence and mortality rates (World) in 2020, World, both sexes, all ages.

According to the latest data from the Global Cancer Observatory (GCO) (Cancer Today (iarc.fr)), in 2020, lung cancer ranked as the third most common cancer globally, with an incidence rate of approximately 22.4 cases per 100,000 population and a mortality rate of around 18% per 100,000 population (Sung et al., 2021) (Figure 1). Non-Small Cell Lung Cancer (NSCLC) represents the predominant subtype of lung cancer, accounting for approximately 85% of cases, and it is characterized by a poor prognosis, with a 5-year survival rate of only 19% (Rodríguez-Abreu et al., 2021; Mithoowani and Febbraro, 2022). Over the past few decades, various treatment modalities, including surgery, chemotherapy, radiation therapy, targeted therapy, and immunotherapy, have been employed in the clinical management of NSCLC (Hopstaken et al., 2021; Miller and Hanna, 2021; Wang et al., 2021; Alduais et al., 2023). Despite the significant advances achieved for these treatment strategies, the development of therapy resistance in NSCLC remains a considerable challenge (Brown et al., 2019; Patel and Weiss, 2020; Muthusamy et al., 2022), thus necessitating the exploration of novel therapeutic approaches for NSCLC.

Ferroptosis is a novel form of programmed cell death (PCD) that has been recently discovered and differs morphologically, biochemically, and genetically from apoptosis, autophagy, and necrotic (Mou et al., 2019; Chen et al., 2021b; Yuan et al., 2021). The presence of ferroptosis in cells is commonly linked to the accumulation of iron, disturbances in fatty acid metabolism, and lipid peroxidation (Ursini and Maiorino, 2020; Xu et al., 2021; Yao et al., 2021), which play crucial roles in initiating ferroptosis (Li and Li, 2020; Zhang et al., 2022). Previous studies have highlighted the significant role of ferroptosis in the pathogenesis of NSCLC, suggesting ferroptosis maybe a potential and novel approach for NSCLC treatment (Liu et al., 2021; Liu et al., 2022). The mediators or signal pathways regarding to ferroptosis in the pathological progression of NSCLC were presented in Table 1.

**TABLE 1:** The mediators or signal pathways regarding to ferroptosis in the pathological progression of NSCLC.

Mediators or signal pathways	Full name	Mechanism	References
FSP1	S100 calcium binding protein A4	FSP1 reduces ubiquinone (CoQ) to ubiquinol (CoQH2). As an antioxidant, CoQH2 inhibits lipid peroxidation and prevents ferroptosis	Bersuker et al. (2019)
Nrf-2/HMOX1	NFE2 like bZIP transcription factor 2/heme oxygenase 1	Acetaminophen sensitizing erastin-induced ferroptosis via modulation of Nrf-2/heme oxygenase-1 signaling pathway in non-small-cell lung cancer	Gai et al. (2020b)
HO-1	heme oxygenase-1	Lysosomal destabilizing drug Siramesine and the dual tyrosine kinase inhibitor Lapatinib induce a synergistic ferroptosis through reduced heme oxygenase-1(HO-1) levels	Villalpando-Rodriguez et al. (2019)
Notch3	notch receptor 3	Notch3 regulates ferroptosis via ROS-induced lipid peroxidation in NSCLC.	Li et al. (2022e)
TP53	tumor protein p53	Upregulation and activation of p53 by erastin-induced reactive oxygen species contribute to cytotoxic and cytostatic effects in A549 lung cancer cells	Huang et al. (2018)
P53RRA	long intergenic non-protein coding RNA 472	P53RRA promoted ferroptosis and apoptosis by affecting transcription of several metabolic genes	Mao et al. (2018)
NFS1	NFS1 cysteine desulfurase	NFS1 undergoes positive selection in lung tumours and protects cells from ferroptosis	Alvarez et al. (2017)
YAP	Yes associated transcriptionalregulator ring finger protein 113A	Intercellular interaction dictates cancer cell ferroptosis via NF2-YAP signaling	Wu et al. (2019)
RNF113A	Lymphoid-specific helicase	The X-linked trichothiodystrophy-causing gene RNF113A links the spliceosome to cell survival upon DNA damage	Shostak et al. (2020)
LCH	serine/threonine/tyrosine kinase 1	EGLN1/c-Myc Induced Lymphoid-Specific Helicase Inhibits Ferroptosis through Lipid Metabolic Gene Expression	Jiang et al. (2017)
STYK1	NFE2 like bZIP transcription factor 2	STYK1/NOK correlates with ferroptosis in non-small cell lung carcinoma	Lai et al. (2019)
Nrf2	nuclear paraspeckle assembly transcript 1	Nrf-2 regulates the sensitivity of human NSCLC cells to cystine deprivation-induced ferroptosis via FOCAD-FAK	Hu et al. (2020a)
NEAT1	long intergenic non-protein coding RNA 336	NEAT1 inhibits acyl-CoA synthetase long chain family member 4 (ACSL4) expression level to promotes ferroptosis sensitivity	Wu and Liu (2021)
LINC00336	microRNA 324	LINC00336 served as an endogenous sponge of microRNA 6852 (MIR6852) to regulate the ferroptosis	Wang et al. (2019)
MIR324	microRNA 4443	MIR324 direct targets GPX4 and reinstates ferroptosis sensitivity in cisplatin-resistant A549/DDP cells	Deng et al. (2021)
MIR4443	microRNA 302a	MIR4443 suppresses cisplatin-induced ferroptosis by modulating expression of apoptosis inducing factor mitochondria associated 2 (AIFM2) in an m6A-dependent manner	Song et al. (2021b)
MIR302A	metallothionein 1D	MIR302A participates ferroptosis process via targeting ferroportin in lung cancer cells	Wei et al. (2021)
MT1DP	epidermal growth factor receptor	MT1DP sensitized A549 and H1299 cells to erastin-induced ferroptosis through downregulation of Nrf-2	Gai et al. (2020a)
EGFR		Activation of EGFR pathway can increase Nrf-2 expression in which upregulates GPX4 expression and inhibits EGFR-tyrosine kinase inhibitor (TKI) -induced ferroptosis	Ma et al. (2021)

In recent years, natural products from traditional herbal medicine have emerged as an increasingly important therapy in the prevention and treatment of NSCLC (Zhang et al., 2018; Wan et al., 2019; Li et al., 2021). Furthermore, there is a growing body of research focusing on the modulation of ferroptosis by natural products for the prevention and treatment of NSCLC (Batbold and Liu, 2021). This article discusses the molecular mechanisms underlying ferroptosis and highlights the mechanisms by which different types of natural products induce ferroptosis to exert anti-cancer effects on NSCLC. The aim is to further provide theoretical support for drug development and treatment strategies in NSCLC.

## 2 Mechanism of ferroptosis

**FIGURE 2:**
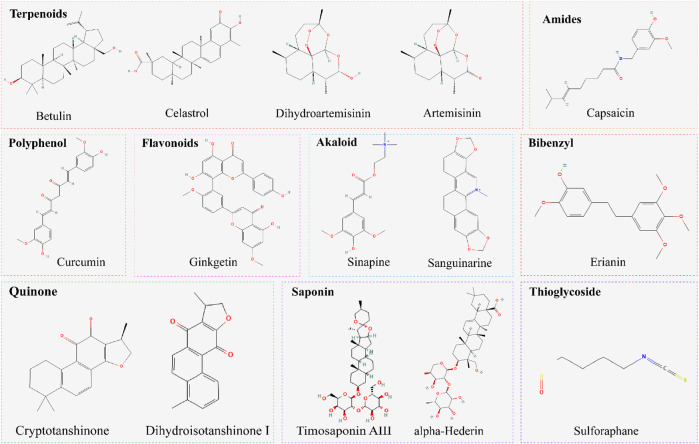
Regulatory mechanism of ferroptosis. General mechanism of ferroptosis associated with System Xc^−^, GPX4, accumulation of iron, and lipid peroxidation.

Ferroptosis represents a distinct form of cell death, which was first proposed by Dixon et al., in 2012 (Dixon et al., 2012). Morphologically, ferroptosis is characterized by mitochondrial shrinkage, mitochondrial membrane rupture, increased membrane density, and reduced or vanished mitochondrial cristae (Xie et al., 2016). Biochemically, lipid peroxidation, iron metabolism, redox homeostasis and fatty acid supply are currently thought to be pivotal to the induction of ferroptosis. (Li et al., 2020; Chen et al., 2021c). The following part provides an overview of the extensively studied mechanisms underlying ferroptosis (Figure 2).

### 2.1 Inhibition of the cysteine-glutamate transporter system Xc^−^ induces ferroptosis

The system Xc^-^ is a transport system involved in the regulation of cellular redox balance and the production of GSH (Liu et al., 2021). It is composed of two subunits: the light chain, known as xCT (SLC7A11), and the heavy chain, known as solute carrier family 3 member 2 (SLC3A2) (Tu et al., 2021). xCT is responsible for the transport of cystine (oxidized form of cysteine) into the cell, while SLC3A2 acts as a chaperone and stabilizes the expression of xCT on the cell surface (Wang et al., 2022). The function of system Xc^-^ is to transport cystine into the cell in exchange for glutamate (Glu) export. Cystine is a disulfide form of the cysteine, which is essential for the synthesis of the antioxidant GSH. GSH helps to neutralize reactive oxygen species (ROS) and protects cells from oxidative damage (Albrecht et al., 2010). In the context of ferroptosis, system Xc^-^ plays a central role in maintaining intracellular redox homeostasis. It imports cystine into the cell, which is subsequently reduced to cysteine. By promoting the availability of cystine, system Xc^-^ supports the synthesis of GSH by catalyzing glutathione synthetase (GSS) and glycine (Gly). Throughout this process, GSH undergoes oxidation to form oxidized glutathione (GSSG). However, GSSG is subsequently converted back to its reduced form, GSH, with the assistance of an enzyme called glutathione reductase (GR). Therefore, inhibition or genetic depletion of system Xc^-^ leads to impaired cystine uptake, reduced GSH synthesis, and increased vulnerability to lipid peroxidation, ultimately promoting ferroptosis (Li F. J. et al., 2022).

### 2.2 Inhibition of GPX4 induce ferroptosis

GPX4, a crucial antioxidant enzyme predominantly localized within cellular organelle membranes, plays a significant role in the elimination of lipid peroxides (Miao et al., 2022). Specifically, it exhibits the capacity to catalyze the reaction between GSH and lipid peroxidation during the process of ferroptosis. This enzyme facilitates the reduction of lipid peroxidation into benign alcohol forms, thereby impeding the buildup of lipid peroxidation (Sui et al., 2018). However, under conditions of inadequate intracellular GSH levels, the functionality of GPX4 becomes hindered, impeding the effective clearance of lipid peroxidation (Xu et al., 2021). During ferroptosis, the inhibition of system Xc^-^ leads to a reduction in GSH synthesis, diminishes the substrate availability for GPX4 and reduces the elimination of lipid peroxidation, ultimately provokes the initiation of ferroptosis (Seibt et al., 2019).

### 2.3 Accumulation of iron

Iron is an essential bio-element within cells, participating in various physiological processes, including oxygen transport (Lipper et al., 2019), DNA synthesis (Lane et al., 2015), and energy production (Hentze et al., 2004). Under certain conditions, iron can also act as catalysts for cell death, promoting the occurrence of ferroptosis (Ma et al., 2022; Anandhan et al., 2023). Ferric ions (Fe^3+^) are imported into the cell from the extracellular space through their binding to transferrin (TF), forming the complex “TF–Fe^3+^–TfR1” with transferrin receptor 1 (TfR1) (Hadzhieva et al., 2014; Basuli et al., 2017). This process involving TF and TfR1 is crucial for the intracellular accumulation of lipid peroxides and the occurrence of ferroptosis. Within the endosome, Fe^3+^ are converted to ferrous ions (Fe^2+^) by ferric reductases such as STEAP3 metalloreductase (Ye et al., 2022). Subsequently, Fe^2+^ are transported from the endosome to the labile iron pool (LIP) via the divalent metal transporter 1 (DMT1) (Aschner et al., 2022). In the cytosol, Fe^2+^ reacts with hydrogen peroxide (H_2_O_2_) through the Fenton reaction, leading to lipid peroxidation and the generation of ROS(Liu et al., 2022). Importantly, various cellular processes that influence iron uptake, storage, utilization, and release can impact cell sensitivity to ferroptosis. For instance, the degradation of ferritin induced by nuclear receptor coactivator 4 (NCOA4) also contributes to ferroptosis promotion (Santana-Codina et al., 2021). Conversely, reduced expression of solute carrier family 40 member 1 (SLC40A1) may result in intracellular Fe^2+^ accumulation, subsequently increase iron-dependent oxidative stress and facilitate ferroptosis (Hao et al., 2021).

### 2.4 Lipid peroxidation

Lipid metabolism plays a vital role in the occurrence of ferroptosis, which is characterized by the accumulation of lipid peroxides resulting from the oxidation of polyunsaturated fatty acids (PUFAs), a class of fatty acids characterized by the presence of multiple double bonds, including omega-3 and omega-6 fatty acids (Christie and Harwood, 2020), which plays essential roles in the composition of cell membranes and participate in numerous physiological processes within cells (Wiktorowska-Owczarek et al., 2015). During the process of lipid peroxidation, several enzymes involved in lipid metabolism act as positive regulators of ferroptosis. One such enzyme is Acyl-CoA synthetase long chain family member 4 (ACSL4), which participates in phospholipid metabolism and facilitates the synthesis of PUFA-CoA from PUFAs like arachidonoyl (AA) and adrenal (AdA), thereby activating PUFAs(Doll et al., 2017). Following ACSL4-driven esterification, lysophosphatidic transferase 3 (LPCAT3) incorporates PUFAs into phospholipids, forming phospholipids containing PUFAs (Reed et al., 2022). Subsequently, ALOX15 oxidizes these PUFA-PLs, generating lipid peroxides and ultimately leading to ferroptosis (Ma et al., 2022).

### 2.5 Others

Ferroptosis can also be regulated by several another protein. Recent publications are summarized in Table 2.

**TABLE 2:** Mediators or modulators of ferroptosis.

Proteins	Full names	Mechanisms	References
Panx 1	Pannexin 1	Panx 1 downregulates lipid peroxidation through the MAPK signal pathway	Su et al. (2019)
VDACs	voltage-dependent amino channels	Generation of mitochondrial ROS and mitochondrial dysfunction	Lipper et al. (2019)
HSPB1	heat shock 27 kDa protein 1	HSPB1 phosphorylation is downregulated and iron-mediated in the production of ROS	Sun et al. (2015)
VDR	Vitamin D receptor	VDR mediates the transcription of GPX4	Hu et al. (2020b)
CARS	Cysteinyl-tRNA synthetase	Involved in the synthesis of GSH	Hayano et al. (2016)
15LO	15-lipoxygenases	Catalyzes the formation of pro-ferroptotic 15-OOH-AA (HpETE)	Stoyanovsky et al. (2019)
PEBP1	Phosphatidylethanolamine-binding protein 1	Restrain the Ras/MEK/ERK cascade	Wenzel et al. (2017)

## 3 Natural products modulating ferroptosis for intervention in NSCLC

**FIGURE 3:**
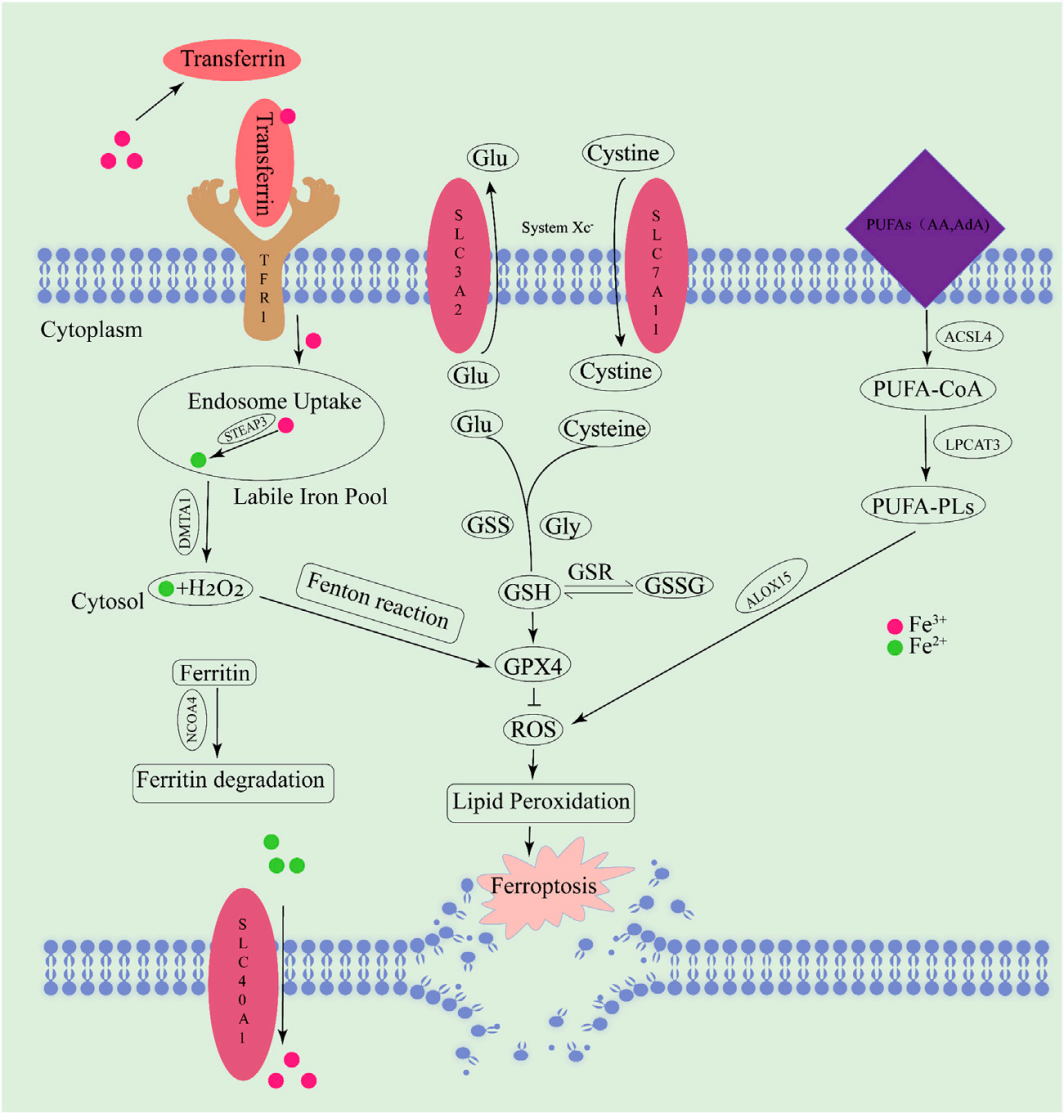
Chemical structures of natural products from traditional Chinese herbal medicine.

**FIGURE 4:**
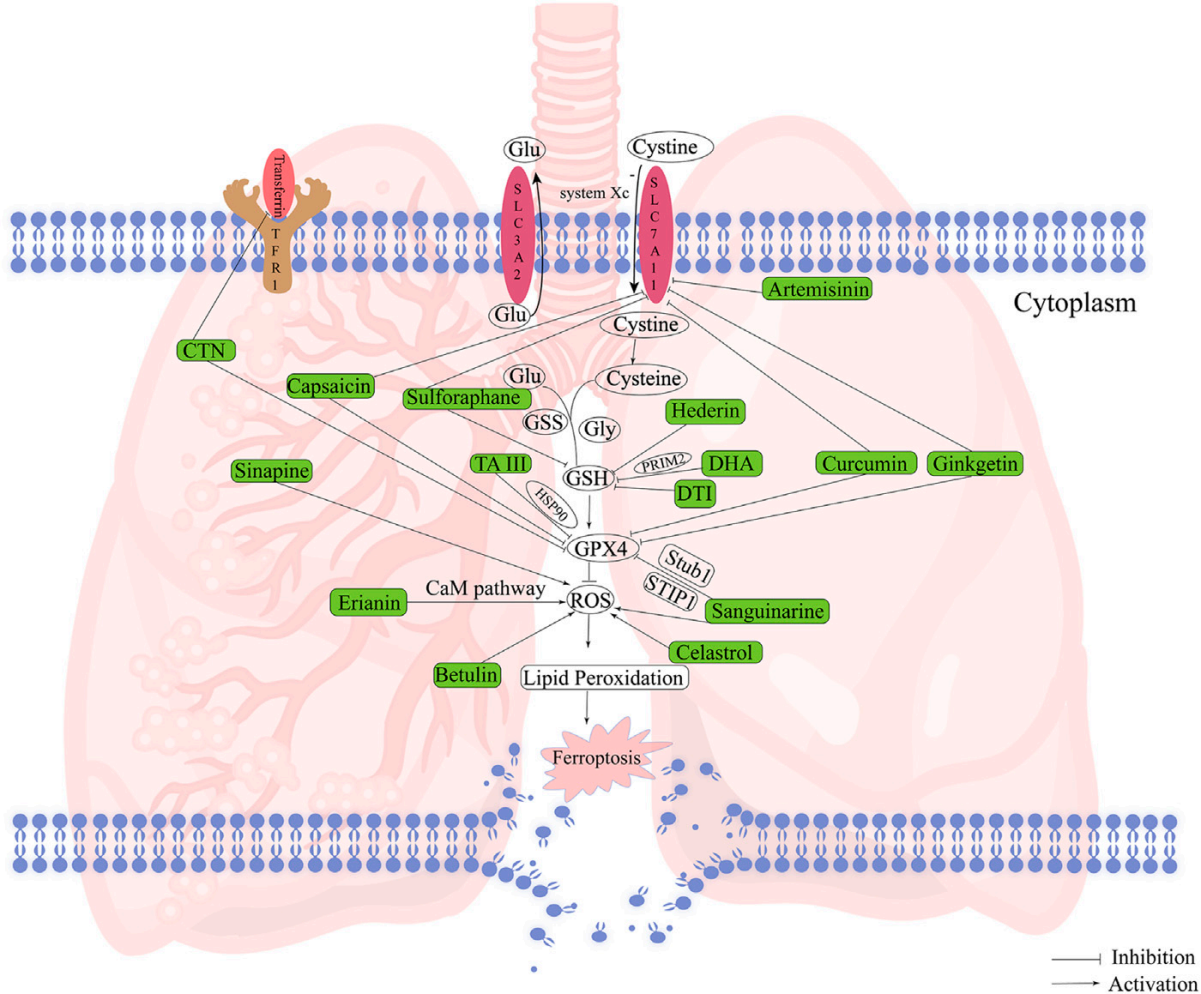
The mechanism of natural products inducing ferroptosis in NSCLC. The activation of ROS or the inhibition of GPX4, GSH, SLC7A11 and transferrin by different natural products can induce ferroptosis to treat NSCLC.

Natural products possess multiple pharmacological activities, particular in the treatment of tumors. Recently, researchers have identified certain natural products that can modulate ferroptosis to exert anti-tumor potential (Yang et al., 2022). Figure 3 provides a compilation of natural products with their sources and chemical formulas, which induce ferroptosis to treat NSCLC. The regulatory targets and mechanisms of these natural products are illustrated in Figure 4 and presented in Table 3.

**TABLE 3:** Natural products inducing ferroptosis in NSCLC.

Natural products	Mechanisms	References	Effectiveness *in vivo*
Timosaponin AIII	Forms a complex with HSP90 and leads to degradation of GPX4	Zhou et al. (2023)	***p < 0.001 *versus* control group in tumor volume (mm^3^)
Curcumin	Downregulation of SLC7A11	Tang et al. (2021)	****p < 0.0001 *versus* control group in tumor volume (mm^3^)
Sanguinarine	Sanguinarine mediated the ubiquitination of GPX4 through Stub1	Xu et al. (2022)	**p < 0.01 *versus* control group in tumor volume (mm^3^)
Erianin	Targeting the CaM signaling pathway and leading to ROS up Ginkgetin reduced the expression of SLC7A11 and GPX4	Chen et al. (2020a)	*p < 0.05 *versus* control group in tumor volume (mm^3^)
Ginkgetin	α-Hederin reduces the expression of GPX2/GSS/GSH	Lou et al. (2021b)	**p < 0.01 *versus* control group in tumor volume (mm^3^)
α-Hederin	Downregulation of GPX4	Wu et al. (2022a)	***p < 0.001 *versus* control group in tumor volume (mm^3^)
Dihydroisotanshinone I	Downregulates SLC7A11 and results in a reduction in GSH	Wu et al. (2021)	*p < 0.05 *versus* control group in tumor metastasis (μm)
Sulforaphane	Downregulation of ferroportin	Jiang et al. (2016), Iida et al. (2021)	**p < 0.01 *versus* control group in tumor volume (mm^3^)
Cryptotanshinone	Downregulates the protein and mRNA levels of Xct	Chen et al. (2014), Popa et al. (2021)	**p < 0.01 *versus* control group in tumor volume (mm^3^)
Artemisinin	Upregulating Xct and downregulating SLC7A11	Chen et al. (2019), Zhang et al. (2021a)	**p < 0.01 *versus* control group in tumor volume (mm^3^)
Sinapine	Inhibiting PRIM2/SLC7A11 Axis	Shao et al. (2022)	**p < 0.01 *versus* control group in tumor volume (mm^3^)
Dihydroartemisinin	Increase the interaction between DRP1 and FIS1	Yuan et al. (2020), Hu et al. (2023)	**p < 0.01 *versus* control group in tumor volume (mm^3^)
Celastrol	Increase ROS accumulation and GSH depletion	Liu et al. (2021a)	***p < 0.001 *versus* control group in tumor volume (mm^3^)
Betulin	Increase the levels of total iron and reduce GSH levels	Li et al. (2022c), Yan et al. (2022)	***p < 0.001 *versus* control group in tumor size (mm^2^)
Capsaicin		Liu et al. (2022b), Deng et al. (2023)	**p < 0.01 *versus* control group in maximal distances of metastasis (μm)

### 3.1 Timosaponin AIII

Timosaponin AIII(TA III) is a steroidal saponin and major active component derived from the traditional Chinese medicinal herb *Schisandra chinensis* (Wang et al., 2023). Timosaponin AIII exhibits various pharmacological activities, including anti-inflammatory (Yuan et al., 2016), anti-oxidant (Jiang et al., 2014), and anti-cancer effects (Chien et al., 2022). Zhou (Zhou et al., 2023) discovered that Timosaponin AIII can inhibit the proliferation and migration of NSCLC cells, induce cell cycle arrest at the G2/M phase, and trigger ROS release and iron accumulation. This process is accompanied by the generation of malondialdehyde (MDA) and the depletion of GSH. Furthermore, it was confirmed that heat shock protein 90 (HSP90) is a direct target of Timosaponin AIII. Timosaponin AIII forms a complex with HSP90, leading to the ubiquitination and degradation of GPX4, ultimately inducing ferroptosis. This study confirms that Timosaponin AIII can play a therapeutic role in NSCLC by inducing ferroptosis.

### 3.2 Curcumin

Curcumin is a naturally occurring compound derived from turmeric (Anand et al., 2007). The curcumin possesses pharmacological properties such as immunomodulation (Gautam et al., 2007), anti-inflammatory (Peng et al., 2021), and anti-cancer activity (Gouda and Bhandary, 2019). In a study conducted by Tang (Tang et al., 2021), characteristic changes associated with ferroptosis were observed in response to curcumin treatment in NSCLC cells. These changes included increased intracellular accumulation of iron and MDA, depletion of GSH, downregulation of SLC7A11 and GPX4 protein levels, negative regulators of ferroptosis, accumulation of lipid peroxidation, curcumin-induced mitochondrial membrane rupture, and reduction of mitochondrial cristae. Furthermore, the curcumin-treated group exhibited increased accumulation of autolysosomes, along with elevated levels of autophagy biomarkers, such as Beclin1 and microtubule-associated protein 1 light chain 3 alpha (LC3). The study also demonstrated that the characteristic changes of ferroptosis were partially reversed when the curcumin-treated NSCLC cells group was treated with CQ (an inhibitor of autophagosome-lysosome fusion) or when the Beclin1 was silenced. In summary, these findings suggest that autophagy contributes to curcumin-induced ferroptosis in NSCLC cells, and inhibiting autophagy can alleviate cellular sensitivity to ferroptosis.

### 3.3 Sanguinarine

Sanguinarine is a natural small-molecule compound isolated from the bloodroot plant (Lou et al., 2021), which possesses anti-bacterial (Zhang et al., 2020), anti-inflammatory (Niu et al., 2012), and anti-cancer properties (Gaziano et al., 2016). Xu (Xu et al., 2022). conducted a study and found that sanguinarine inhibited the proliferation of NSCLC cells in a dose-dependent and time-dependent manner. In xenograft tumor animal models, sanguinarine effectively suppressed the growth and metastasis of NSCLC cells. Furthermore, it was discovered that sanguinarine induced intracellular accumulation of iron, increased levels of ROS and MDA, and reduced GSH levels. Sanguinarine mediated the ubiquitination and degradation of GPX4 through stress induced phosphoprotein 1 (STIP1) and U-Box containing protein 1 (Stub1), thereby triggering ferroptosis in NSCLC cells. Furthermore, overexpression of GPX4 partially restored the proliferative and invasive inhibitory effects of sanguinarine on NSCLC cells by suppressing ferroptosis. In conclusion, sanguinarine inhibits the growth and metastasis of NSCLC cells by regulating the Stub1/GPX4-dependent iron-dependent cell death pathway.

### 3.4 Erianin

Erianin, a natural benzylisoquinoline compound extracted from *Dendrobium* (Li et al., 2023), exhibits anti-cancer activity by inhibiting cell proliferation, inducing apoptosis (Xu et al., 2021), and autophagy (Chen et al., 2020) in various cancers such as cervical cancer (Li et al., 2018), colorectal cancer (Miao et al., 2023), prostate cancer (Trapika et al., 2021), and breast cancer (Xie et al., 2021). Chen (Chen et al., 2020) found that erianin arrested NSCLC cells in the G2/M phase, thereby inhibiting cell proliferation and metastasis. Further investigations revealed that erianin treatment induced accumulation of ROS, consumption of GSH, and occurrence of lipid peroxidation in NSCLC cells. However, the results were reversed by ferroptosis inhibitor. Calmodulin (CaM), a major endogenous calcium-regulating protein, plays a crucial role in regulating L-type voltage-dependent calcium channels, which are important for calcium and iron transport (Zeng et al., 2023). This study confirmed that erianin targeted the CaM signaling pathway, leading to ROS accumulation and upregulation of iron by modulating the calcium-CaM pathway, thereby inducing ferroptosis in NSCLC cells.

### 3.5 Ginkgetin

Ginkgetin is a natural flavonoid compound derived from the *Ginkgo biloba*. It exhibits pharmacological activities such as neuroprotection (Singh et al., 2019), cardiovascular protection (Silva and Martins, 2022), anti-oxidant (Li et al., 2022d), and anti-inflammatory effects (Li et al., 2022b). Additionally, research has shown that it exerts anti-cancer effects by inhibiting cell proliferation, angiogenesis, and inducing apoptosis in tumor cells (DeFeudis et al., 2003; Ye et al., 2007; Bai et al., 2015; Han et al., 2016; Kim et al., 2021). Lou (Lou J. S. et al., 2021) conducted a study and found that the combination of Ginkgetin with cisplatin enhanced the cytotoxicity against NSCLC cells. This combination treatment increasesthe accumulation of iron and the occurrence of lipid peroxidation. Further investigations revealed that Ginkgetin reduced the expression of SLC7A11 and GPX4, decreased the GSH/GSSG ratio, increased ROS formation, and reduced the activity of the Nrf-2/HO-1 signaling pathway. These actions collectively induced ferroptosis in NSCLC cells. These results suggest that the combination of Ginkgetin and cisplatin reduces NSCLC development by inducing ferroptosis.

### 3.6 Hederin

Hederin belongs to a class of saponin compounds found in the Ginkgo biloba plant, which belongs to the family *Ginkgoaceae* (Jeong et al., 2019). Numerous studies have indicated that α-Hederin has anti-tumor functions (Belmehdi et al., 2023). For instance, in colorectal cancer cells (Sun et al., 2019), α-Hederin inhibits the epithelial-mesenchymal transition induced by interleukin-6 and the activity of the JAK2/STAT3 signaling pathway, thereby suppressing cell migration and invasion. In gastric cancer cells (Wang et al., 2020), the combination of α-Hederin and cisplatin promotes apoptosis in gastric cancer cells through mitochondria-related apoptotic pathways. Wu (Wu et al., 2022) discovered that α-Hederin inhibits the proliferation and invasion of NSCLC cells in a dose-dependent manner both *in vitro* and *in vivo*. Subsequent proteomics, metabolomics, and high-throughput sequencing confirmed that α-Hederin treatment reduces the expression of GSH peroxidase 2 (GPX2) and GSS, inhibits the synthesis of GSH, disrupts the GSH redox system. After the administration of the ferroptosis inhibitor of ferrostatin-1, the study observed a partial restoration of α-Hederin-induced cell death. Meanwhile, ferrostatin-1 treatment recovered α-Hederin-induced disturbance in mitochondrial membrane potential. In summary, α-Hederin could induces ferroptosis in the treatment of NSCLC.

### 3.7 Dihydroisotanshinone I

Dihydroisotanshinone I (DTI), a diterpenoid compound belonging to the tanshinone class, is extracted from the medicinal herb *Salvia miltiorrhiza* (Hsu et al., 2021). *Salvia miltiorrhiza* has been widely used in traditional Chinese medicine and possess diverse pharmacological activities and therapeutic potential (XD et al., 2019; He et al., 2024). Research studies have demonstrated that DTIDTI exhibits anti-oxidant (Ip et al., 2002), and anti-cancer properties (Wu et al., 2017; Lin et al., 2019). Wu (Wu et al., 2021)have shown that DTI inhibits the growth of A549 cells and H460 cells via inducing ferroptosis. The underlying mechanism involves the downregulation of GPX4 protein and GSH levels, accumulation of MDA,ROS, leading toand lipid peroxidation and finally induction of ferroptosis in NSCLC.

### 3.8 Sulforaphane

Sulforaphane belongs to the class of compounds known as isothiocyanates and is predominantly found in vegetables of the Brassicaceae family, particularly in cruciferous vegetables such as cauliflower, cabbage, and broccoli (Vanduchova et al., 2019). Extensive research has demonstrated that sulforaphane exerts its beneficial effects through multiple mechanisms, including the activation of intracellular antioxidant enzymes (Ishida et al., 2021), modulation of cellular signaling pathways (Zhang et al., 2022c), and anti-cancer activities (Russo et al., 2018). Studies conducted by Yuko Iida (Iida et al., 2021) have shown that sulforaphane significantly inhibits the growth of NSCLC cells, and this growth inhibition can be reversed by ferroptosis inhibitors offerrostatin-1. Furthermore, treatment of NSCLC cells with sulforaphane leads to an increase in iron and ROS levels, as well as collection of lipid peroxidation products, all of which can be attenuated by ferroptosis inhibitors. Subsequent investigations revealed that sulforaphane specifically downregulates the expression of the SLC7A11, resulting in a reduction in GSH accumulation. Collectively, Sulforaphane inhibits the growth of NSCLC cells via inducing ferroptosis.

### 3.9 Cryptotanshinone

Cryptotanshinone (CTN) is a diterpenoid monomer and a lipophilic component extracted from the dried roots and rhizomes of the traditional Chinese herb *S. miltiorrhiza* (Li et al., 2021; Zhang et al., 2023). CTN has been proven to possess various biological activities, including antioxidant (Guo et al., 2022), anti-tumor (Dalil et al., 2022), antibacterial (Zhong et al., 2021), and anti-inflammatory effects (Wu et al., 2020). Li (Popa et al., 2021) discovered that CTN effectively suppresses NSCLC cells invasion, proliferation and tumorigenesis. The treatment of CTN resulted in increased iron accumulation within the cells and decreased the expression levelof GPX4 protein. Furthermore, CTN elicited an upregulation of Cytoglobin, a protein known to induce ferroptosis, while downregulats ferroportin expression. Moreover, study demonstrated that CTN induces iron-dependent lipid peroxidation by inhibiting the function of TfR1. These data suggest that the induction of ferroptosis in NSCLC cells, achieved by increasing iron accumulation, Cytoglobin, and iron-dependent lipid peroxidation, or by downregulating the expression levels of ferroportin and GPX4, may be an important mechanism through which CTN attenuates NSCLC.

### 3.10 Artemisinin

Artemisinin is a sesquiterpene lactone isolated from the *Artemisia annual* (Ma et al., 2020). It is widely used for the treatment of malaria (Talman et al., 2019). Apart from its well-known role in treating malaria, artemisinin has been reported in numerous studies to possess additional pharmacological activities, including anti-schistosomiasis (Pérez del Villar et al., 2012),anti-cancer (Kiani et al., 2020),anti-inflammation (Yuan et al., 2019),anti-virus (Wani et al., 2021). Zhang (Zhang et al., 2021) discovered that artemisinin downregulates the protein and mRNA levels of xCT. Furthermore, artemisinin upregulates the mRNA level of TfR1, Therefore, Zhang hypothesis that Artemisinin may induces ferroptosis in NSCLC. Consequently, the cell death caused by artemisinin can be partially reversed by N-Acetyl-L-cysteine (NAC), a ROS scavenger, and ferrostatin-1, a ferroptosis inhibitor. The findings demonstrate that the inhibitory effect of artemisinin on NSCLC cells is at least partially attributed to the induction of ferroptosis.

### 3.11 Sinapine

Sinapine belongs to the class of compounds known as phenethylamines particularly in abundance in sources such as barley, mustard seeds, peas, and rapeseeds (Dang et al., 2023). It exhibits numerous pharmacological activities, such like antioxidant (Yates et al., 2019), anti-inflammatory (Li et al., 2019), and anti-cancer activities (Guo et al., 2014). In the realm of cancer research, Sinapine has shown promise as an anti-cancer agent, displaying inhibitory effects on various cancer cells, including breast cancer (Guo et al., 2016) and colorectal cancer (Yang et al., 2023). Notably, Shao (Shao et al., 2022) found that Sinapine play the anti-tumor effects on NSCLC cells. Induce ferroptosis by increasing intracellular ferrous iron, lipid peroxidation, and ROS in NSCLC cells. Also, treatment with Sinapine upregulates transferrin and transferrin receptor, and inhibits either of them attenuated the ferroptosis induced by Sinapine. Additionally, Sinapine treatment led to a p53-dependent downregulation of SLC7A11. Furthermore, Sinapine also play the inhibition role in the growth of NSCLC *in vivo*. In conclusion, the findings highlight that Sinapine could be a promising therapeutic approach via triggers ferroptosis in NSCLC.

### 3.12 Dihydroartemisinin

Dihydroartemisinin (DHA) is a derivative of artemisinin, which is a compound extracted from the *Artemisia annua* plant (Dai et al., 2021). DHA exhibits its antimalarial activity by rapidly and effectively clearing the malaria parasite from the bloodstream (Hanboonkunupakarn and White, 2022). In addition to its antimalarial properties, DHA has been demonstrated anti-inflammatory (Yang et al., 2022),anti-cancer (Bai et al., 2021) and immunomodulatory (Gao et al., 2020) properties. In DNA replication, the protein encoded by the DNA primase subunit 2 (PRIM2) gene plays a critical role. PRIM2 functions by catalyzing the synthesis of RNA primers, which act as the starting points for DNA synthesis (Wei and Lozano-Durán, 2023). Yuan’s study (Yuan et al., 2020) revealed that DHA reduced the expression of PRIM2, and silencing PRIM2 mimicked the inhibitory effects of DHA on cell proliferation and colony formation, while promoting cell death in NCSLC cells. Additionally, the study found that DHA treatment and the absence of PRIM2 led to a series of ferroptosis characteristic in NSCLC cells. Mechanistically, the combination of DHA treatment and the absence of PRIM2 decrease the level of GSH, increasecellular lipid ROS and MDA levels, as well as downregulats SLC7A11 and β-catenin expressions in NCSLC cells.

### 3.13 Celastrol

Celastrol is a prominent bioactive compound extracted from the root bark of Tripterygium wilfordii, a plant belonging to the *Celastraceae* family (Xu et al., 2021). It falls within the class of pentacyclic triterpenoids, possessing a triterpene framework and exhibiting noteworthy biological activity (Li et al., 2022f), such like anti-cancer (Xu et al., 2023), anti-inflammatory effect in liver fibrosis (Wang et al., 2020), and potential pharmacological treatment of obesity (Liu et al., 2015). In a study conducted by Liu(Liu et al., 2021), it was observed that the combination of erastin,a ferroptosis inducer, and celastrol induced cell death in NSCLC cells at concentrations that were not toxic individually. The co-treatment with celastrol and erastin resulted in promotion of ROS generation, disturbance of mitochondrial membrane potential, augmentation of the interaction between dynamin-related protein 1 (DRP1) and mitochondrial fission, mitochondrial 1 (FIS1), and stimulation of mitochondrial fission. Of these, the above results suggest that Celastrol may be a natural compound that effectively induces ferroptosis.

### 3.14 Betulin

Betulin is a natural triterpene compound that occurs in the bark of specific tree species, including white and silver birch trees (Demets et al., 2022). The emerging evidence has shown that Betulinpossesses various advantageous properties, including anti-inflammatory (Tuli et al., 2021), anti-oxidant (Günther et al., 2021), and anti-cancer (Wang et al., 2017). Yan (Yan et al., 2022) found that betulin in combination with Gefitinib exhibited antagonistic effects on cellular viability on NSCLC cells of A549 and H460. However, the ferroptosis inhibitors of ferrostatin-1, liproxstatin-1 and deferoxamine can completely rescue the viability of A549 and H460 after treatment of betulin in combination with Gefitinib. Moreover, in order to confirm whether ferroptosis contributes to the death under the treatment of betulin in combination with gefetinib, the author performed a series of experiments and found that combination induced ROS accumulation, lipid peroxidation, and GSH depletion. The expression of SLC7A11, GPX4 and ferritin heavy chain 1 (FTH1), negative regulators of ferroptosis, was decreased under the combination treatment of betulin and gefetinib. Whereas, the positive regulatory protein of ferroptosis heme oxygenase 1(HO-1) was increased. , Therefore, Betulin may be a potential therapeutic agent for NSCLC via inducing ferroptosis.

### 3.15 Capsaicin

Capsaicin, a natural compound present in chili peppers, in fruits of the capsicum genus like cayenne peppers and jalapenos (Chapa-Oliver and Mejía-Teniente, 2016), has attracted attention for its potential health benefits (Sharma et al., 2013). Research suggests that capsaicin may contributes to improved cardiovascular health (Munjuluri et al., 2021) and possess anti-microbial properties (Goci et al., 2021). In a study conducted by Liu(Liu et al., 2022), it was observed that capsaicin exhibited significant inhibitory effects on the proliferation of NSCLC cells. Mechanismlly, capsaicin increased the levels of total iron and ferrous ions, while reducing GSH levels in the treated cells compared to the control group. Additionally, both mRNA and protein levels of SLC7A11 and GPX4 showed significant decreases in NSCLC cells treated with capsaicin compared to the control group. In summary, the treatment potential of capsaicin in NSCLC cells lies in its ability to induce ferroptosis.

### 3.16 Chinese medicine and preparations

In addition, several studies have indicated that certain Chinese medicine and preparations possess the potential therapeutic ability to stimulate ferroptosis to exhibit potential benefits in the treatment of NSCLC. *Hedyotis diffusa* (HD), a species of flowering plant in the family *Rubiaceae,* is a traditional Chinese herbal medicine, which exhibits numerous pharmacological activities, including antioxidant (Lu et al., 2000), anti-inflammatory (Hung et al., 2022), and anti-cancer (Wu et al., 2022). It contains various bioactive compounds, including iridoids, flavonoids, triterpenoids, and phenolic acids, which are believed to contribute to its medicinal effects (Zhang et al., 2021). Huang (Huang et al., 2022)found that HD can inhibit NSCLC cells growth and induce characteristics of ferroptosis, including increase in mitochondrial membrane density, shrunken mitochondria, and decline of cristae. Moreover, HD increase cellular Lipid ROS, the Fe^2+^ fluorescence intensity and MDA levels. Mechanically, HD-induced ferroptosis in lung adenocarcinoma cells may be related to the voltage dependent anion channel 2/3(VDAC2/3) pathway, (Yang et al., 2020),a group of specific channel proteins, facilitates the exchange of metabolites and ions across the outer mitochondrial membrane and may regulate mitochondrial functions. Furthermore, HD exerts its regulatory effects on the BCL2 apoptosis regulator (Bcl2)/BCL2 associated X, apoptosis regulator (Bax) protein complex, thereby modulating the functional dynamics of VDAC2/3. This modulation results in the activation of VDAC2/3 channels, facilitating the translocation of ions and facilitating the intracellular accumulation of ROS. Above all, HD could induce ferroptosis via Bcl2 inhibition to promote Bax regulation of VDAC2/3 to attentus the NSCLC cells growth. Zhao (Zhao et al., 2022) showed that Fuzheng Kang’ai (FZKA) decoction significantly suppressed the expression of GPX4 and system Xc^−^ and conducted a reduction in the GSH/GSSG ratio to induce ferroptosis in NSCLC treatment. Importantly, the induction of ferroptosis in NSCLC cells by FZKA decoction was significantly reversed when GPX4 was overexpressed. These findings were further confirmed *in vivo* animal model, validating the observed effects of FZKA on ferroptosis in NSCLC cells.

## 4 Conclusion and prospects

Ferroptosis is a novel form of cell death that distinguishes itself from apoptosis, necroptosis, and autophagy. Multiple evidences have indicated the significant role of ferroptosis in regulating tumor cell growth and drug resistance, making it a potential new target for anti-tumor interventions (Chen et al., 2021a; Song et al., 2021; Wang et al., 2022). Therefore, ferroptosis inducers hold great promise as highly prospective agents for cancer diagnosis and therapeutic intervention, and they are also of significant importance in the development of anti-cancer drugs (Lei et al., 2022). Here, we have summarized the characteristic ferroptosis inducers and their main anti-cancer mechanisms in Supplementary Table, aiming to provide support for the clinical development of anti-cancer drugs.

The current study summarized the introduction and mechanism of ferroptosis. The mechanism of ferroptosis mainly involves four processes: GPX4, system Xc^−^, iron metabolism, and lipid peroxidation. In the field of NCSLC research, the ferroptosis as a novel cellular death mechanism has gathered significant attention in recent years (Zhang et al., 2022b; Zhao et al., 2023). We summarized relevant targets or pathways of ferroptosis in NCSLC therapy as shown in Table 1. Additionally, natural products have made certain progress in the prevention and treatment of NCSLC by regulating ferroptosis process. However, the investigation into the modulation of ferroptosis in NSCLC using natural products is currently in its initial exploratory phase, with certain limitations evident in existing research studies. Firstly, most studies have only explored the molecular mechanisms by which natural product from traditional Chinese medicine induces ferroptosis through a single pathway. Nevertheless, traditional Chinese medicine formulas and patent medicines widely used in clinical practice possess the characteristics of “multiple components, multiple targets, and multiple effects” (Yan et al., 2022). In future research, we should explore the molecular mechanisms of traditional Chinese medicine in regulating ferroptosis from multiple pathways and perspectives, construct “components-targets/pathways-disease” pharmacological network, and conduct relevant clinical trials.

Secondly, natural products are typically complex mixtures with diverse chemical structures and compositions, which increases the complexity of studying their pharmacological activities and tissue specificity (Xia et al., 2022). These complexities may make it difficult to accurately assess the absorption, distribution, metabolism, and excretion properties of drugs. Based on this, we need to strengthen the exploration of the potential of nanotechnology (Kaur et al., 2022), encapsulation (Linh et al., 2022), and targeted delivery systems (Gorain et al., 2022) to improve the pharmacokinetics and tissue specificity of natural products.

Thirdly, certain natural products may cause adverse reactions in the digestive system, such as nausea, vomiting, diarrhea, gastrointestinal discomfort, etc. (Menniti-Ippolito et al., 2008; Vasudeva et al., 2012; Zorzela et al., 2021; Andrade et al., 2022). Therefore, the importance of conducting controlled clinical trials should be emphasized to evaluate the safety of treatment methods based on natural products for NSCLC patients.

Finally, it is also necessary to consider combining natural products with other treatment modalities, including chemotherapy drugs such as sulfasalazine (Lay et al., 2007), sorafenib (Groenendijk et al., 2015), zalcitabine (McNeill and Wilson, 2007), and cisplatin (Kryczka et al., 2021), which all can be purposed to induce ferroptosis, to improve the efficiency of natural product-based treatments for NSCLC. We believe that with the progress ferroptosis of research, new effective strategies for the treatment of NSCLC can be provided.

## Figures and Tables

**Table:** 

**ACSL4**	Acyl-CoA synthetase long chain family member 4
**AA**	arachidonoyl
**AdA**	adrenal
**CTN**	Cryptotanshinone
**CaM**	Calmodulin
**CoQ**	Coenzyme Q
**DTI**	Dihydroisotanshinone I
**DMT1**	divalent metal transporter 1
**DHA**	Dihydroartemisinin
**DRP1**	dynamin-related protein 1
**Fe** ^**3+**^	Ferric ions
**Fe** ^**2+**^	ferrous ions
**FZKA**	Fuzheng Kang’ai
**FIS1**	fission 1 protein
**GSH**	glutathione
**GCO**	Global Cancer Observatory
**GSSG**	oxidized glutathione
**GR**	glutathione reductase
**Gly**	glycine
**GSS**	glutathione synthetase
**GPX4**	glutathione peroxidase 4
**glutamate**	Glu
**GPX2**	GSH peroxidase 2
**HD**	Hedyotis diffusa
**HSP90**	heat shock protein 90
**LPCAT3**	lysophosphatidic transferase 3
**MDA**	malondialdehyde
**Nrf-2**	NFE2 like bZIP transcription factor 2
**NCOA4**	nuclear receptor coactivator 4
**NAC**	N-Acetyl-L-cysteine
**NSCLC**	Non-Small Cell Lung Cancer
**PCD**	programmed cell death
**PUFAs**	polyunsaturated fatty acids
**PRIM2**	DNA primase subunit 2
**ROS**	reactive oxygen species
**Stub1**	U-Box containing protein 1
**SLC3A2**	solute carrier family 3 member 2
**SLC40A1**	solute carrier family 40 member 1
**TA III**	Timosaponin AIII
**TfR1**	transferrin receptor
**VDAC**	voltage-dependent anion channel
**xCT**	SLC7A11
