# Supplementary material for: Induction of ferroptosis by natural products in non-small cell lung cancer: a comprehensive systematic review
Source: Front Pharmacol. 2024 May 1;15:1385565. doi: 10.3389/fphar.2024.1385565 (PMC11094314; doi:10.3389/fphar.2024.1385565)
Supplement: Supplementary file 1 [file Table1.docx]

**Supplementary Table 1.** **Ferroptosis inducer and anticancer applications**

| **Ferroptosis inducer** | **Tumor Types** | **Reference** |
| --- | --- | --- |
| Auranofin | Neuroblastoma | (Floros et al., 2021) |
| Erastin | Fibrosarcoma | (Kim et al., 2020) |
| Imidazole ketone Erastin | Diffuse large B cell lymphoma | (Zhang et al., 2019) |
| Salicylazosulfapyridine | Lung cancer | (Su et al., 2020) |
| Dyclonine | Squamous cell carcinoma | (Okazaki et al., 2018) |
| Sorafenib | Renal cell carcinoma | (Fishman et al., 2015) |
| RAS selective lethal 3 | Glioma | (Wang et al., 2019) |
| Bortezomib | Prostatic cancer | (Cramer et al., 2017) |
| Fluvastatin | Breast cancer | (Garwood et al., 2010) |
| Withaferin A | Neuroblastoma | (Hassannia et al., 2018) |
| Lapatinib | Breast cancer | (Wu et al., 2022) |
| Siramesin | Breast cancer | (Ma et al., 2016) |

**[Reference]**

Cramer, S.L., Saha, A., Liu, J., Tadi, S., Tiziani, S., Yan, W., et al. (2017). Systemic depletion of L-cyst(e)ine with cyst(e)inase increases reactive oxygen species and suppresses tumor growth. *Nat Med* 23(1)**,** 120-127. doi: 10.1038/nm.4232.

Fishman, M.N., Tomshine, J., Fulp, W.J., and Foreman, P.K. (2015). A systematic review of the efficacy and safety experience reported for sorafenib in advanced renal cell carcinoma (RCC) in the post-approval setting. *PLoS One* 10(4)**,** e0120877. doi: 10.1371/journal.pone.0120877.

Floros, K.V., Cai, J., Jacob, S., Kurupi, R., Fairchild, C.K., Shende, M., et al. (2021). MYCN-Amplified Neuroblastoma Is Addicted to Iron and Vulnerable to Inhibition of the System Xc-/Glutathione Axis. *Cancer Res* 81(7)**,** 1896-1908. doi: 10.1158/0008-5472.CAN-20-1641.

Garwood, E.R., Kumar, A.S., Baehner, F.L., Moore, D.H., Au, A., Hylton, N., et al. (2010). Fluvastatin reduces proliferation and increases apoptosis in women with high grade breast cancer. *Breast Cancer Res Treat* 119(1)**,** 137-144. doi: 10.1007/s10549-009-0507-x.

Hassannia, B., Wiernicki, B., Ingold, I., Qu, F., Van Herck, S., Tyurina, Y.Y., et al. (2018). Nano-targeted induction of dual ferroptotic mechanisms eradicates high-risk neuroblastoma. *J Clin Invest* 128(8)**,** 3341-3355. doi: 10.1172/jci99032.

Kim, H., Lee, J.H., and Park, J.W. (2020). Down-regulation of IDH2 sensitizes cancer cells to erastin-induced ferroptosis. *Biochem Biophys Res Commun* 525(2)**,** 366-371. doi: 10.1016/j.bbrc.2020.02.093.

Ma, S., Henson, E.S., Chen, Y., and Gibson, S.B. (2016). Ferroptosis is induced following siramesine and lapatinib treatment of breast cancer cells. *Cell Death Dis* 7(7)**,** e2307. doi: 10.1038/cddis.2016.208.

Okazaki, S., Shintani, S., Hirata, Y., Suina, K., Semba, T., Yamasaki, J., et al. (2018). Synthetic lethality of the ALDH3A1 inhibitor dyclonine and xCT inhibitors in glutathione deficiency-resistant cancer cells. *Oncotarget* 9(73)**,** 33832-33843. doi: 10.18632/oncotarget.26112.

Su, Y., Zhao, B., Zhou, L., Zhang, Z., Shen, Y., Lv, H., et al. (2020). Ferroptosis, a novel pharmacological mechanism of anti-cancer drugs. *Cancer Lett* 483**,** 127-136. doi: 10.1016/j.canlet.2020.02.015.

Wang, X., Lu, S., He, C., Wang, C., Wang, L., Piao, M., et al. (2019). RSL3 induced autophagic death in glioma cells via causing glycolysis dysfunction. *Biochem Biophys Res Commun* 518(3)**,** 590-597. doi: 10.1016/j.bbrc.2019.08.096.

Wu, X., Sheng, H., Zhao, L., Jiang, M., Lou, H., Miao, Y., et al. (2022). Co-loaded lapatinib/PAB by ferritin nanoparticles eliminated ECM-detached cluster cells via modulating EGFR in triple-negative breast cancer. *Cell Death Dis* 13(6)**,** 557. doi: 10.1038/s41419-022-05007-0.

Zhang, Y., Tan, H., Daniels, J.D., Zandkarimi, F., Liu, H., Brown, L.M., et al. (2019). Imidazole Ketone Erastin Induces Ferroptosis and Slows Tumor Growth in a Mouse Lymphoma Model. *Cell Chem Biol* 26(5)**,** 623-633.e629. doi: 10.1016/j.chembiol.2019.01.008.
